# Supplementary material for: High quality genome assemblies reveal evolutionary dynamics of repetitive DNA and structural rearrangements in the Drosophila virilis sub-group
Source: bioRxiv. 2023 Aug 14:2023.08.13.553086. Preprint. [Version 1] doi: 10.1101/2023.08.13.553086 (PMC10462019; doi:10.1101/2023.08.13.553086)
Supplement: Supplement 4 [file media-4.pdf]

Table S1 Strains sequenced in this study.

Strain number	Shortform	Species	Accession
15010-1051.51	vir51	*D. virilis*	PRJNA548201
15010-1051.52	vir52	*D. virilis*	PRJNA548201
15010-1051.86	vir86	*D. virilis*	PRJNA548201
15010-1051.47	vir47	*D. virilis*	PRJNA548201
15010-1051.49	vir49	*D. virilis*	PRJNA548201
15010-1051.85	vir85	*D. virilis*	PRJNA548201
15010-1051.08	vir08	*D. virilis*	PRJNA548201
15010-1051.00	vir00	*D. virilis*	PRJNA548201
15010-1051.118	vir118	*D. virilis*	PRJNA548201
15010-1051.48	vir48	*D. virilis*	PRJNA548201
15010-1051.87	vir87/GDvir*	*D. virilis*	PRJNA548201
Toyama15		*D. virilis*	This study, PRJNA981214
Vzzp01 (f)		*D. virilis*	This study, PRJNA981214
vww8		*D. virilis*	This study, PRJNA981214
England1430 (f)		*D. virilis*	This study, PRJNA981214
England1431		*D. virilis*	This study, PRJNA981214
amMK1012		*D. americana*	PRJNA548201
amCI0518		*D. americana*	PRJNA548201
amCI0515		*D. americana*	PRJNA548201
amG96*		*D. americana*	PRJNA548201
amCI0538		*D. americana*	PRJNA548201
amMK0738		*D. americana*	PRJNA548201
amSB		*D. americana*	PRJNA548201
amML975		*D. americana*	PRJNA548201
15010-1031.14	nov14/GDnov*	*D. novamexicana*	PRJNA548201
15010-1031.04	nov4	*D. novamexicana*	PRJNA548201
15010-1031.08	nov8	*D. novamexicana*	PRJNA548201
15010-1031.12	nov12	*D. novamexicana*	PRJNA548201
15010-1031.13	nov13	*D. novamexicana*	PRJNA548201

* Genome strains sequenced with PacBio and assembled.

Table S2 High confidence Y protein coding genes that with functional annotation.

species	seqid	gene	product
D. ame	tig00000523	*MSTRG.16118*	Dynein heavy chain 2, axonemal
D. ame	tig00000545	*MSTRG.16125*	Transcription-associated protein 1
D. ame	tig00000640	*MSTRG.16192*	Dynein heavy chain 5, axonemal
D. ame	tig00000640	*MSTRG.16193*	Retrovirus-related Pol polyprotein from transposon 412
D. ame	tig00000656	*MSTRG.16208*	Retrovirus-related Gag polyprotein from transposon gypsy
D. ame	tig00000656	*MSTRG.16217*	Dynein heavy chain 2, axonemal
D. ame	tig00000686	*MSTRG.16222*	Dynein regulatory complex subunit 3
D. ame	tig00003077	*MSTRG.16623*	Dynein heavy chain 8, axonemal
D. nov	NW_020824857.1	*LOC115767635*	dynein beta chain, ciliary-like
D. nov	NW_020824875.1	*LOC115768126*	dynein beta chain, ciliary
D. nov	NW_020824893.1	*LOC115768466*	protein Hook homolog 3-like
D. nov	NW_020824893.1	*LOC115768469*	protein Hook homolog 3-like
D. nov	NW_020824896.1	*LOC115768531*	dynein heavy chain 5, axonemal-like
D. nov	NW_020824896.1	*LOC115768532*	dynein heavy chain 5, axonemal-like
D. nov	NW_020824912.1	*LOC115768695*	dynein heavy chain 2, axonemal-like
D. nov	NW_020824944.1	*LOC115768949*	DBF4-type zinc finger-containing protein 2 homolog
D. nov	NW_020824946.1	*LOC115768961*	histone H2A-like
D. nov	NW_020824946.1	*LOC115768962*	dynein heavy chain 2, axonemal-like
D. nov	NW_020824970.1	*LOC115769090*	dynein heavy chain 8, axonemal-like
D. nov	NW_020824971.1	*LOC115769091*	cytosolic non-specific dipeptidase-like
D. nov	NW_020824975.1	*LOC115769109*	cilia- and flagella-associated protein 58-like
D. nov	NW_020824996.1	*LOC115769151*	dynein regulatory complex subunit 3-like
D. vir	NW_022587449.1	*LOC116650070*	transmembrane protein 19-like
D. vir	NW_022587439.1	*LOC26530771*	dynein beta chain, ciliary
D. vir	NW_022587438.1	*LOC26530795*	dynein heavy chain 8, axonemal
D. vir	NW_022587438.1	*LOC26531380*	dynein heavy chain 5, axonemal
D. vir	NW_022587449.1	*LOC26531539*	dynein heavy chain 8, axonemal
D. vir	NW_022587459.1	*LOC26531779*	dynein beta chain, ciliary
D. vir	NW_022587459.1	*LOC26531779*	dynein beta chain, ciliary
D. vir	NW_022587442.1	*LOC26531848*	histone H1.2-like
D. vir	NW_022587474.1	*LOC26531899*	dynein heavy chain 2, axonemal
D. vir	NW_022587425.1	*LOC6635996*	cytosolic non-specific dipeptidase
D. vir	NW_022587442.1	*LOC6636766*	DBF4-type zinc finger-containing protein 2 homolog
D. vir	NW_022587441.1	*LOC6636891*	protein Hook homolog 3
D. vir	NW_022587521.1	*LOC6637063*	dynein heavy chain 2, axonemal

Table S3 Summary of number and length of ITR arrays in virilis group genomes

Species	Chromosome	Number_arrays	Avg_array_length	Average number of arrays per chromosome	Average length of arrays per chromosome	[illegible]
D. ame	2	208	7357.09	342.6	5489.27	
D. ame	3	342	5897.51			
D. ame	4	537	4216.83			
D. ame	5	310	3761.77			
D. ame	6	26	3265.15			
D. ame	X	316	6213.15			
D. vir	2	380	1666.87	370.4	1845.538	
D. vir	3	297	2611.85			
D. vir	4	366	2081.19			
D. vir	5	303	1556.63			
D. vir	6	0	0			
D. vir	X	506	1311.15	316	4633.41	
D. nov	2	146	6541.23			
D. nov	3	329	4969.43			
D. nov	4	501	4039.77			
D. nov	5	331	3572.31			
D. nov	6	277	1169.4			
D. nov	X	312	4044.31			

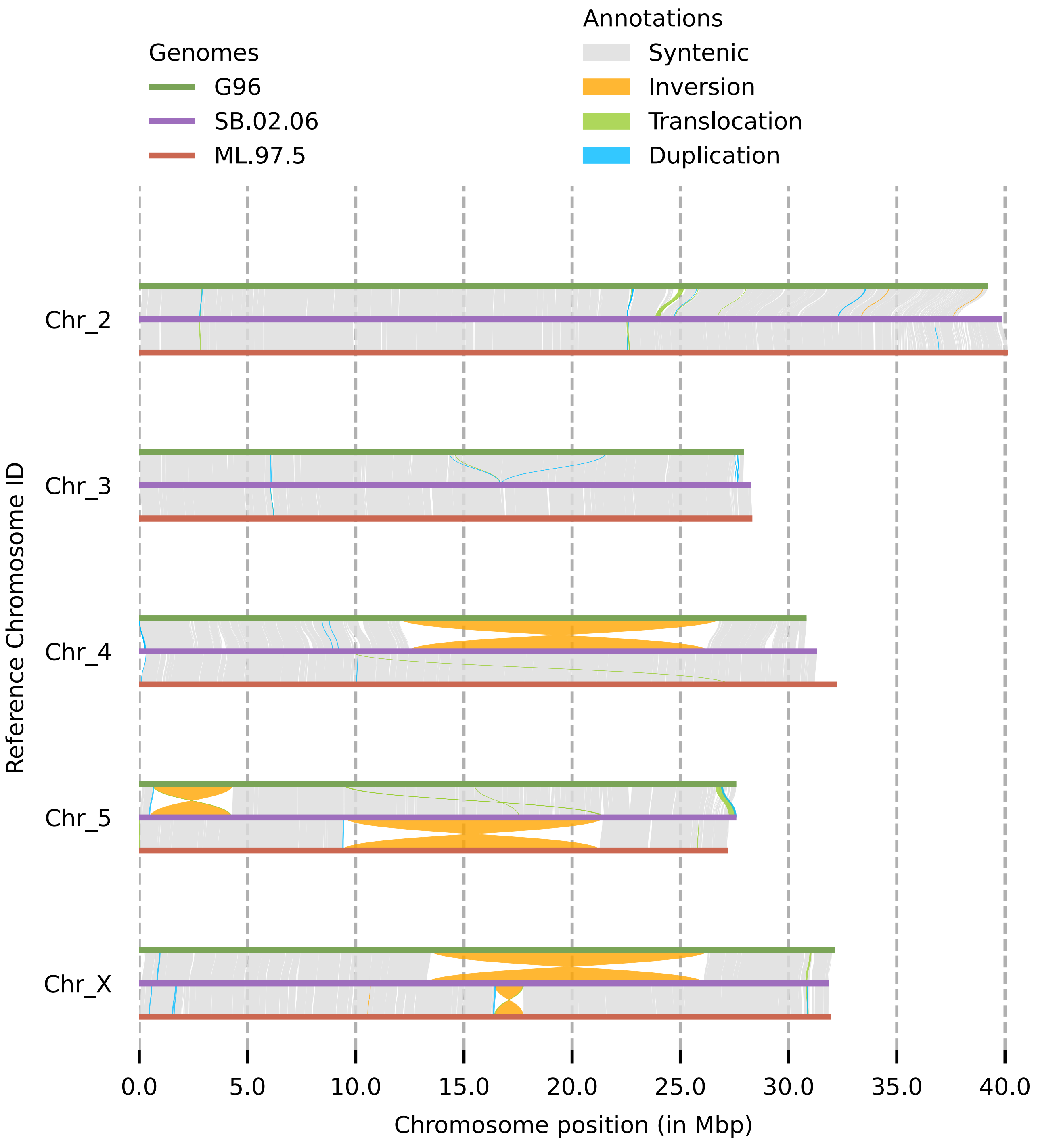

Figure S1 Whole chromosome alignment of the three *D. americana* genomes showing the main inversion differences between the three strains.

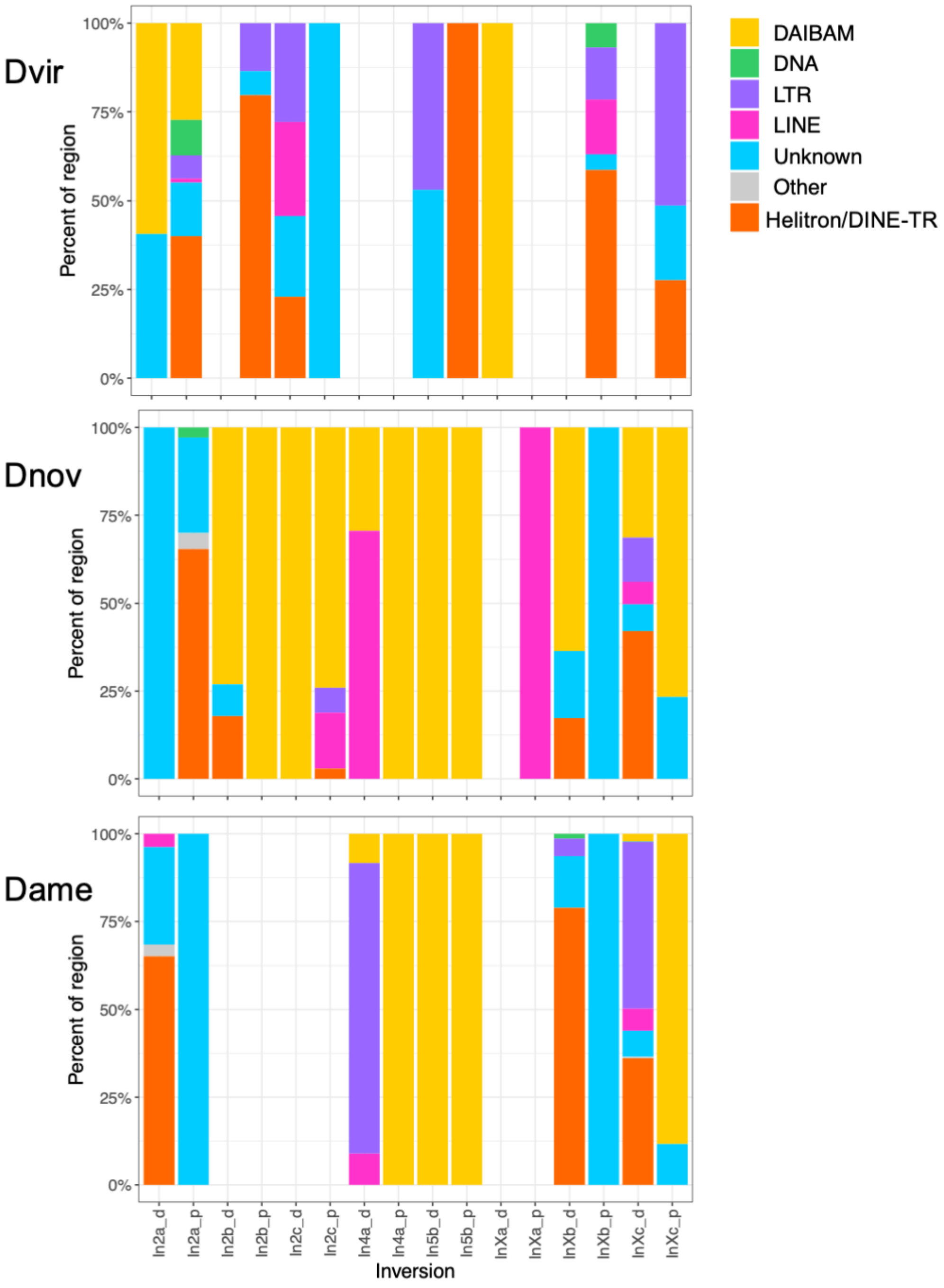

Figure S2 TE content in regions flanking predicted inversion breakpoints in each species. We intersected the repeat annotation from RepeatMasker with predicted breakpoints. Bars are colored by the TE subclass; bars are absent if there were no repetitive elements in the breakpoint region. If TEs were present, then the regions were extended to the TE's called end point, normalizing each to 100%.

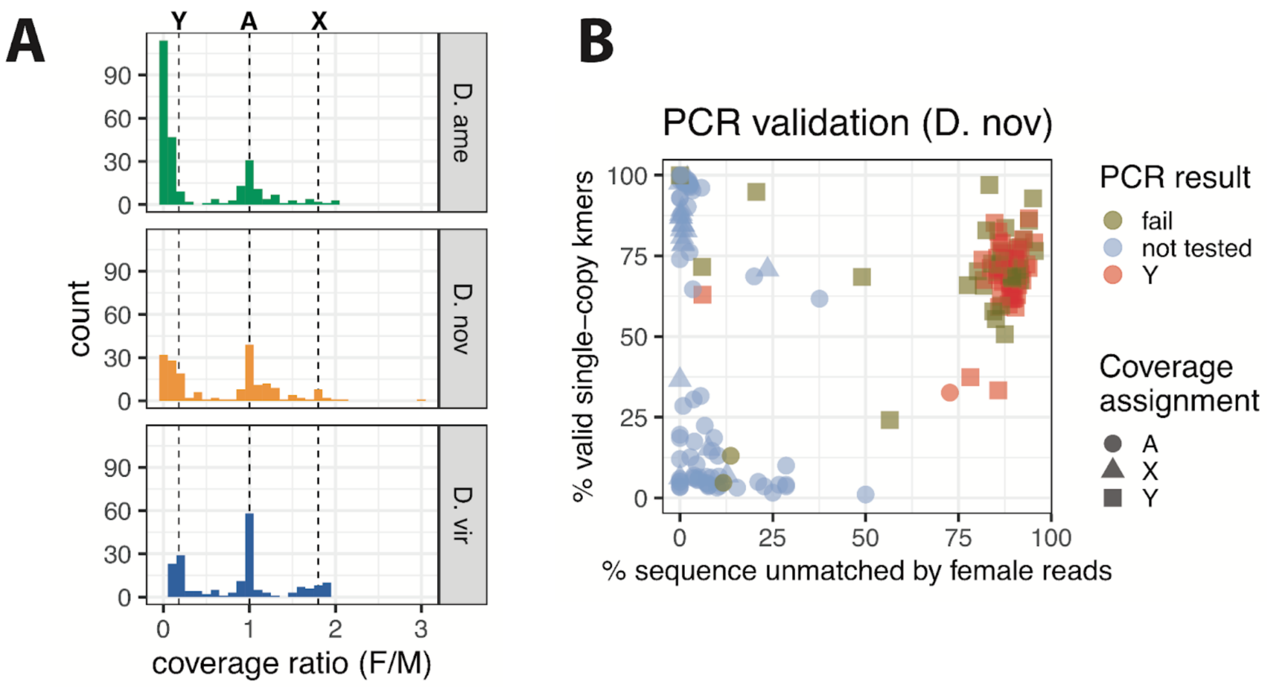

Figure S3 (A) Contig coverage distribution in the three scaffolded assemblies. The dotted lines indicate the expected coverage ratio for the Y chromosome, autosomes and the X chromosome. (B) Genomic contigs plotted based on *k*-mer matches of female-derived reads (*x*-axis) and the percentage of single-copy *k*-mers (*y*-axis) in *D. novamexicana* with the PCR validation results indicated. Contigs that amplified in the male sample but not the female sample are classified as "Y", whereas those that amplify in both sexes are classified as "failed" to validate.

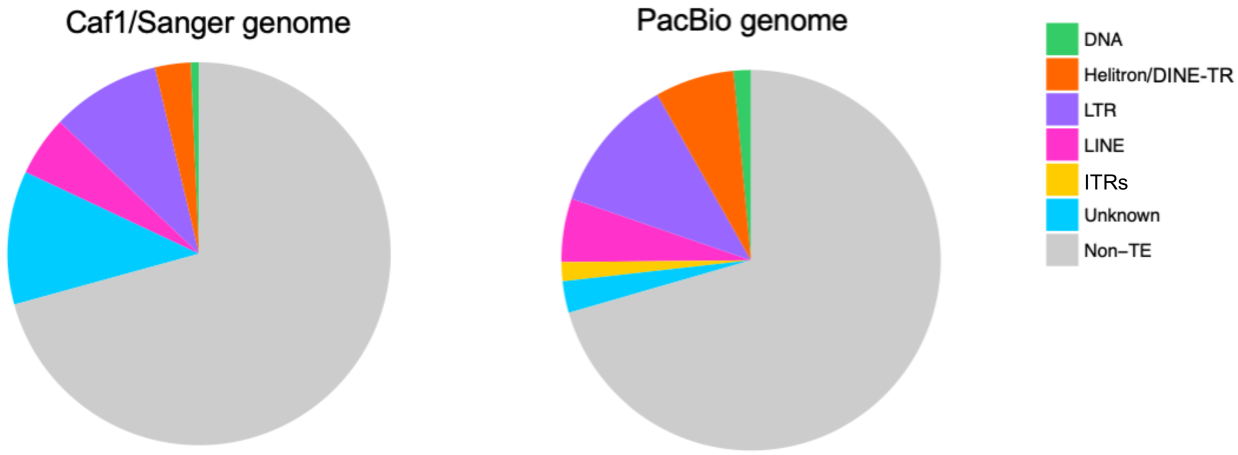

Figure S4 Transposable element composition in the Caf1/Sanger assembly compared to our new PacBio assembly.

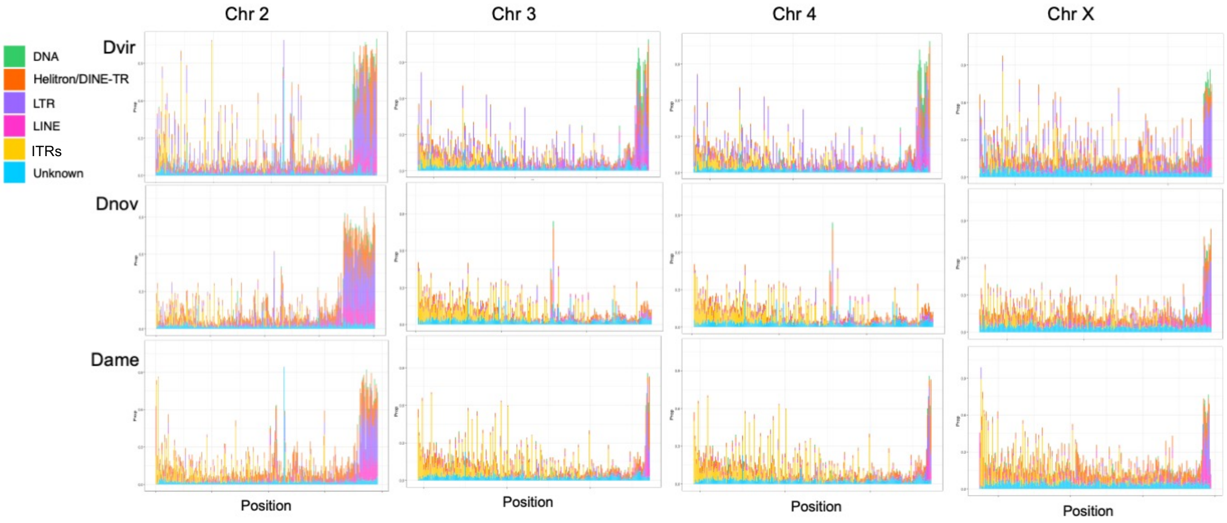

Figure S5 TE density in by subclass 100 kb windows across chromosomes 2, 3, 4, and X (Chr 5 is included in the main text) in the virilis clade.

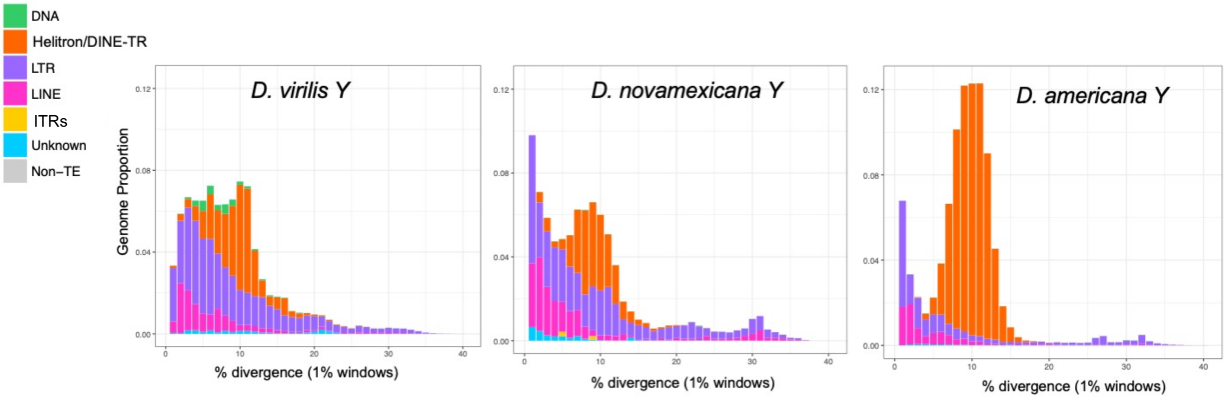

Figure S6 Divergence landscape of the Y chromosome in *D. virilis*, *D. novamexicana*, and *D. americana*.
